# A qualitative study on measuring patient‐centered care: Perspectives from clinician‐scientists and quality improvement experts

**DOI:** 10.1002/hsr2.140

**Published:** 2019-11-04

**Authors:** Sadia Ahmed, Andrea Djurkovic, Kimberly Manalili, Balreen Sahota, Maria J. Santana

**Affiliations:** ^1^ Department of Community Health Sciences, Cumming School of Medicine University of Calgary Calgary Alberta Canada; ^2^ Department of Student Accessibility Services, Student and Enrolment Services University of Calgary Calgary Alberta Canada; ^3^ Department of Paediatrics, Cumming School of Medicine University of Calgary Calgary Alberta Canada

**Keywords:** patient‐centered care, person‐centered care, qualitative research, quality improvement, quality indicators

## Abstract

**Background and aims:**

Patient‐centered care (PCC) benefits patients, health‐care providers, and health‐care systems by providing delivery of care that addresses patient values and needs while improving provider experiences, and by decreasing health‐care expenditure. To improve PCC, health‐care systems need to measure it. Recently, we developed a PCC framework that is evidence based and patient informed. The purpose of this study was to gather the perspective of clinician‐scientists and quality improvement experts regarding the PCC domains included in the framework. Their perspectives were used to refine these domains, which ultimately will inform the development of PCC quality indicators.

**Methods:**

Participants were recruited via expert and snowball sampling. Semi‐structured interviews were conducted with clinician‐scientists and quality improvement experts from Canada, the United States, and the United Kingdom from October 2017 to January 2018. With the use of an interview guide developed using the PCC framework, interviews were audio recorded and transcribed for a thematic analysis using NVivo qualitative data analysis software. Inductive thematic analysis was used to identify themes and subthemes.

**Results:**

Sixteen semi‐structured interviews were conducted, which included four clinician‐scientists and 12 quality improvement experts. Twelve of the participants were from Canada, three from the United Kingdom, and one from the United States. From the thematic analysis, three major themes were identified: (a) measurability of PCC, (b) practical considerations for implementing measurement, and (c) policy and practice implications. Participants discussed barriers and recommendations to improve and increase the clarity of the PCC domains in health system reporting, resulting in several future directions to refine and target specific PCC domains.

**Conclusion:**

Clinician‐scientists and quality improvement experts provided key recommendations for the measurement of PCC. The perspectives of key stakeholders in PCC measurement will inform strategies for the implementation and uptake of patient‐centered quality indicators in health‐care systems. The views of these key experts can lay the foundation for the development of standardized measures of PCC, to ensure monitoring and improvement of PCC.

## 1 INTRODUCTION

Patient‐centered care (PCC) is a model of care guided foremost by the needs and values of patients.^1^ Patient‐centered care is an increasingly well‐recognized and highly sought‐after model of care, reaching the height of its prominence in a report published by the Institute of Medicine, which listed PCC as one of the six most important dimensions of high‐quality care^1^, ^2^ and defined PCC as care that is “respectful of and responsive to individual patient preferences, needs, and values, and ensures that patient values guide all clinical decisions.”^1^, ^3^

Previous research has found that PCC has the potential to improve health outcomes^4^, ^5^, ^6^, ^7^, ^8^, ^9^, ^10^, ^11^, ^12^, ^13^, ^14^, and benefits health‐care systems and health‐care providers. Practice that goes against principles of PCC, such as failure to consider the patient's wishes in decisions related to care, has been associated with accusations of malpractice.^15^, ^16^ When a provider fails to consider a patient's needs and values, there is a risk for miscommunication. Additionally, health‐care systems benefit from PCC in decreasing patients' length of stay, minimizing the need for unnecessary testing and procedures, and decreasing the cost per case, ultimately improving the efficiency of care.^5^, ^9^, ^14^

Numerous frameworks have been developed for PCC, such as Mead and Bower's conceptual framework for patient‐centeredness^17^ and Scholl et al's integrative model of patient‐centeredness.^18^ However, most PCC frameworks have not focused on the practical implementation of PCC in health‐care systems. Additionally, there is currently no systematic approach in place to measure the quality of the provision of PCC.^16^, ^19^, ^20^, ^21^ Patient‐centered quality indicators (PC‐QIs) should be developed to measure PCC in a standard manner.

### 1.1 How to practice person‐centered care: A conceptual framework

A PCC framework was developed in collaboration with a patient partner, following a narrative review of the literature synthesizing evidence, recommendations, and best practice from existing frameworks, and case studies on the delivery of PCC.^22^ This framework categorizes PCC into three components and a total of 13 domains (Appendix S1). The Donabedian model for health‐care improvement^23^ was utilized to classify domains into the categories of structure, process, and outcome. The first component, *structure*, involves seven domains that focus on PCC at foundational and organizational levels, such as creating a PCC culture, codesigning the development and implementation of educational programs, and supporting a workforce committed to PCC.^22^ The second component, *process*, involves four domains that focus on PCC from a patient and health‐care provider level, such as cultivating communication and respectful and compassionate care.^22^ The final domain, *outcome*, involves two PCC domains (access to care and patient‐reported outcomes [PROs]) that focus on outcomes related to access and patient reports.^22^ The PCC framework will be used to guide the identification, development, and classification of PC‐QIs and will serve as a cognitive tool to ensure that the PC‐QIs are consistent with the key dimensions of PCC.

While this framework provides a theoretical and empirical basis for measuring PCC, there is a need to engage clinicians and quality improvement experts, as the users of these measures, for monitoring and improving the quality of care. In particular, it is critical to ensure that the proposed areas of measurement are seen to be relevant to their work and feasible to implement in practice. Hence, the purpose of this study is to elicit the opinions of clinician‐scientists and quality improvement experts regarding the proposed domains of PCC that will inform the development of PC‐QIs. Specific objectives include the following:

1. to explore the views of clinician‐scientists and quality improvement experts regarding proposed domains of PCC, and
2. to gain an understanding of current practices and opportunities for measurement of PCC at a health‐care system level.

## 2 METHODS

This qualitative study used semi‐structured interviews to explore the views of clinician‐scientists and quality improvement experts regarding PCC measurement, acceptability, and feasibility. The semi‐structured interview guide was developed in collaboration with the PC‐QI research team at the University of Calgary and is based on the 13 domains of PCC in the conceptual framework.^22^ The interview guide aimed to refine the proposed definition of a PC‐QI, the feasibility of the PCC domains, and impacts on policy, and to identify potential barriers and facilitators to implement, measure, and report PCC domains (Appendix S2). The interview guide was first piloted with local members of the PC‐QI research team who were not involved in this project and was amended based on subsequent discussions between members of the research team.

Participants were identified through expert referral within the existing network of collaborations in previous PCC‐related research and snowball sampling. This existing network of collaborations includes key stakeholders in PCC and quality improvement across Canada and internationally (United States and United Kingdom). These collaborators include researchers from the University of Calgary, Alberta Health Services, provincial health quality councils in Canada, and the Canadian Institute for Health Information, as well as the National Health Service in the United Kingdom and the Picker Institute Europe, the Centre for Person‐centred Care at the University of Gothenburg (Sweden), and the Patient‐Centered Outcomes Research Institute (PCORI, USA). Incorporating participants from Canada, the United States, and England provided a broader understanding of how PCC is conceptualized and measured in different western countries. Participants outside of Alberta were interviewed to enrich our understanding of how PCC measurement is done in different jurisdictions, to provide insight on opportunities for measurement. Clinician‐scientists and quality improvement experts were invited to participate in this study. Twenty‐two experts were approached, and 16 agreed to participate (six participants could not participate due to time). It is often advised that if participants hold expertise in the research area of the given study, then a smaller sample size is required to achieve saturation.^24^, ^25^ Participants were emailed an invitation letter to voluntarily participate in a 30‐minute interview either in person or over the phone. After accepting the interview time, participants were emailed the PCC framework before the interview. Face‐to‐face or one‐on‐one phone interviews were conducted, with an average interview length of 30 minutes (range 20‐50 min). Interviews were conducted by authors M.S., PhD, and A.D., BHSc, both women with qualitative research background. At the time of the study, A.D. was a Bachelor of Health Science student, and M.S. is a patient and family–centered care scientist. The authors of this paper conducted this study to inform the development of PCC quality indicators and to improve monitoring and evaluation of PCC in health‐care systems.

The University Health Research Ethics Board approved this study (REB 15‐2846). Informed consent was obtained from all participants, and all data were anonymized using numbers. The data were stored on password‐protected computers of the researchers, limiting individual identifiers by only including the role and location of the participants.

Interview audio files were transcribed verbatim and imported into NVivo (version 11.4) qualitative software for primary data analysis. First, transcripts were analyzed for codes, with phrases within transcripts referring to specific topics, questions, actions, or perceptions^24^, ^25^, ^26^ by two researchers. Once coding was completed, peer debriefing allowed both researchers to reach a consensus on the interpretation of the findings.

To conduct a thematic analysis, codes were organized based on repetition, material relating directly to research questions, or similarities and differences between participants, as per Ryan and Bernard's^27^ suggestions for thematic analysis. In an open coding process, key phrases were organized into codes or topics of discussion. Codes were condensed and organized into subthemes under higher‐order themes with the research team, with key quotations provided to support subthemes. Inductive thematic saturation was achieved with the interviews, defined as no additional codes or themes identified during data analysis.^28^

### 2.1 Trustworthiness measures

The quality of qualitative research is often assessed using trustworthiness measures.^24^ Participants were asked only open‐ended questions regarding the PCC domains. The current study also sought to enhance dependability by ensuring that all phases of the research process were recorded carefully and by utilizing other members of the research team as auditors of the research process.^24^ Records of recruitment processes, interview transcripts, and data analysis decisions were carefully managed and made accessible to all members of the research team. Periodic team meetings allowed the research team to provide input regarding the research process, enhancing the dependability of the study.

## 3 RESULTS

We conducted 16 interviews with clinician‐scientists and quality improvement experts between October 2017 and January 2018, six of them face to face and 10 via phone. The roles of the participants included project lead in performance measurement and patient‐centered care medical home director, and physician specialties were emergency medicine, respirology, and family medicine. Participant characteristics are summarized in Table 1.

**Table 1:** Participant characteristics

Characteristic	N
Country of residence	
Canada	12 (AB = 1; ON = 1)
United States	1
United Kingdom	3
Role	
Clinician‐scientist	4 (pediatric emergency medicine, respirology, primary care, and nephrology)
Quality improvement experts	
PCC researcher	9
Data expert	3

Abbreviations: AB, Alberta; ON, Ontario; PCC, patient‐centered care.

Three overall themes were identified: (a) measurability of PCC, (b) practical considerations for implementing measurement, and (c) policy and practice implications.

### 3.1 Measurability of PCC

Participants' overall conceptualization of person‐centered care were aligned with the domains of the PCC framework such as codesigning educational programs for PCC, cultivating communication, and engaging patients in managing their care. Participants discussed the applicability of Donabedian framework for monitoring PCC, distinguishability of PCC domains, challenges in measuring subjective domains, and suggestions for the improvement of the PCC framework.

#### 3.1.1 Applicability of the Donabedian framework

Participants commented on the applicability of the Donabedian framework for PCC measurement. Most participants noted the Donabedian framework of structure, process, and outcome to be useful for organizing PCC domains and for monitoring quality of care.

P_12(QI expert)_: The focus on the reliance on the Donabedian framework is useful. It allows it to integrate it into a comprehensive framework in relation to the specific domains.

A clinician‐researcher described how they distinguished between structure and process domains, indicating that structure domains such as “creating a PCC culture” and “educational programs for PCC” were tangible, foundational domains that can be enacted by the inclusion of process domains such as “cultivating communication,” “being respectful,” and “engaging patients.”

#### 3.1.2 Distinguishability of PCC domains

Some participants expressed concerns regarding the specificity of the domains. Many of the domains were perceived as difficult to distinguish from one another and requiring too much clarification, influencing the framework's ease of access. For example, one clinician‐researcher did not understand how notions of communication and culture are separate, although these two concepts exist as separate domains in the PCC framework. Some participants also offered comments for specific domains within the PCC domains framework regarding feasibility, clarity, and their incorporation into existing health‐care systems. For example, the domain “integration of care” was described as unclear and, thus, requires further clarification in future phases of the PC‐QI project.

P_1(PCC researcher)_: … I don't know if it means integration or if you're going to define integration to include transitions of care. Make it more specific or have a separate domain.

#### 3.1.3 Challenges in measuring subjective domains

Some participants also discussed the notion of a “soft domain” and the difficulties in measuring such subjective domains. Patient‐centered care researchers used the term soft domain to refer to domains that are subjective in nature, often referring to “Creating a PCC Culture” as an example of a soft domain.

P_1(Clinician‐researcher)_: Softer pieces about communication and responsiveness, the ones that mean the most to families are going to be the most difficult [to measure].

Numerous suggestions were provided to improve the PCC domains framework. Some participants suggested the inclusion of a measure of provider experience, as it often affects patient experience, adding valuable insight into the quality of PCC.

P_2(Data expert)_:I think we need to get provider feedback at the same time as patient feedback. The clinical care system needs to work for providers … .

Participants suggested the development of standard measures and evaluation tools to measure these domains. Measurement of PCC domains will allow for the standardization of PCC. Some participants identified patient‐reported experience measures (PREMs) and PRO measures (PROMs) as an effective way of capturing patient feedback on care. One physician was concerned about the subjective nature of PROMs and PREMs, introducing bias in measurement.

### 3.2 Practical considerations for implementing measurement

#### 3.2.1 Feasibility of implementation

The main concerns raised by most participants were regarding the number of potential indicators derived from the PCC domains and their feasibility for implementation. Participants mentioned that too many PCC domains and too many indicators may overwhelm health‐care systems, thus hindering meaningful changes in policy and practice.

P_1(Clinician‐scientist)_: The reality is you start to overwhelm people and if you look at every single one of these no one is going to look at that dashboard.

P_14(Clinician‐scientist)_: I would think that these would be feasible but it might be 5–10 years before you actually are able to get these integrated … the caveat is time.

#### 3.2.2 Stakeholder engagement necessary

Some participants also emphasized the need for stakeholder and community engagement in implementing the domains of PCC and, ultimately, designing PC‐QIs. One clinician‐researcher stated that moving PCC domains into practice in Canada depends on vigorous inter‐professional collaboration and community engagement, for instance, bringing together different health‐care professionals in the same room to discuss implementation of PCC. This participant believed that the confidence of stakeholders in the creation of PCC measures is a prerequisite for receptiveness once PC‐QIs are disseminated.

#### 3.2.3 Reporting of measures/indicators

Participants discussed strategies for the reporting of measures and indicators for PCC, such as the inclusion of annual performance dashboards, which could monitor quality indicators and provide yearly reports. The monitoring of PCC through annual performance dashboards would allow health‐care systems to understand the causes of suboptimal performance and effectively improve in the future.^29^ Some participants considered the dashboards to be a feasible way of monitoring PCC as they are already integrated into health‐care systems. However, they were concerned with potential measurement fatigue and overwhelming already busy medical professionals and health‐care systems.

P_6(Data expert)_: I firmly believe that if we pick a couple of key things and focus on improving those, we're going to go a lot further a lot faster … .

All participants highlighted the importance of the 13 PCC domains. Clinician‐scientists suggested that domains that are measured directly from patients themselves are most effective and most difficult to dismiss.

P_9(PCC Researcher)_: So if you were able to get these indicators validated by large numbers of patients, that would be really important. Because it would be really hard for people (clinicians) to say, “I don't accept this.” The answer would be, well too bad if you don't accept it, because you serve the patients and this is what they want.

There were discussions around the concept of “one‐size fits all” solutions when using a single set of PC‐QIs between different levels of the health‐care system. A QI expert from England emphasized the need to specify a target audience for annual performance dashboards and to modify PC‐QIs depending on the target audience. This researcher stated that, from personal experience, national indicators are mainly appropriate for policy makers and governments, whereas individual providers are often more interested in lower‐level indicators that come directly from patients.

P_9(PCC researcher)_: Who's looking at it? What is their understanding? How are they using it? A lot of that is underpinned by: what do they understand about the purpose of it?

### 3.3 Policy and practice implications

#### 3.3.1 Potential for change

Participants discussed potential implications for policy and practice when monitoring and disseminating PC‐QIs. Participants generally perceived the PCC domains as a powerful tool to evaluate the performance of health‐care systems. The extent to which the PCC domains will impact policy and practice is contingent on collaboration with accreditation boards, such as Accreditation Canada, which was highlighted during the interviews by Alberta clinician‐scientists. One clinician‐scientist mentioned the College of Family Physicians Canada council, which audits physicians on their adherence to standards of practice. This participant indicated that the PCC domains will have a more significant impact if integrated into the auditing practices of such authoritative bodies. Another clinician‐scientist also emphasized the potential benefits of using incentives to gain influence on policy and practice.

P_7(Clinician‐scientist)_: They have to be at risk of losing funding, or having the opportunity to gain something out of it … it needs to be marketed and there need to be consequences to different players.

The implementation of a value‐based purchasing program to reward institutions that perform well on measures of PCC was a suggestion by a quality improvement expert from the United States. Specifically, the expert discussed the Hospital Consumer Assessment of Healthcare Providers and Systems (HCAHPS) survey, administrated through a value‐based purchasing program in the United States that allocates funding from the Centers for Medicare & Medicaid Services to hospitals. Clinician‐scientists also suggested that consequences of poor performance on PC‐QIs must be proximal and culturally appropriate to drive behavior, taking the form of individual appeals to ethics or monetary incentives, depending on the target audience.

#### 3.3.2 Defining quality indicator

In order to measure person‐centered care using quality indicators, there needs to be clear guidelines on what constitutes a PC‐QI. Participants discussed the need for a standard definition of PC‐QIs and offered suggestions to the existing definition developed by the PCC research team. The definition of a PC‐QI proposed by our research team was “the unit of measurement of healthcare system performance, that quantifies what matters to patients and families, and to any individual who is in contact with healthcare services,” based on a literature review conducted by Santana et al.^22^ Participants provided suggestions to ensure that the PC‐QI definition captures multiple levels of care, patient experiences, and actual—as well as potential—contact with the health‐care system. Given the suggestions from participants, our research team revised the definition to “… the unit of measurement of healthcare system, *individual, or organizational* performance, that quantifies *the experiences* of patients and *families and what is perceived as important* to patients and families, and to any individual with healthcare needs *or needs for contact* with healthcare services.”

## 4 DISCUSSION

This study elicits the opinions of clinician‐scientists and quality improvement experts regarding the PCC domains included in our previously proposed framework.^22^ These domains will inform the development of the PC‐QIs as key metrics of the PCC model. It is important to note that the PCC domains were described as important dimensions to measure and monitor PCC. Our findings suggest that clinician‐scientists and quality improvement leaders recognize the value of developing and implementing PC‐QIs from the PCC framework, in driving and supporting PCC.

The findings highlighted several points to take into consideration while measuring and monitoring PCC. First, there is a need to consider the stakeholder's confidence in the measures at hand. Our study addressed this consideration by utilizing stakeholder engagement to include the opinions of PC‐QI end users in the development of PC‐QIs. Additionally, most participants highlighted the importance about the applicability of the PCC domains to a multitude of potential target audiences (for example, patients, the public, quality improvement experts, and health‐care providers), each with varying understandings of improvement in the quality of care. Furthermore, to ensure confidence and acceptability of the measures, clarity in distinguishing the different domains of PCC is crucial to the success and widespread acceptance of PCC measurement. Engaging stakeholders is a key process in the development of PCC measures. A potential strategy for engaging stakeholders is implementing “champions” of PCC within each level of the health‐care system.^30^ A PCC champion would be an individual who understands and embodies the principles of PCC within an institution or organization and mediates local problems in adopting PC‐QIs, to effectively implement PC‐QIs within their workforce.

Additional findings suggest that the PCC domains do not adequately capture the importance of the provider perspective in measuring and implementing PCC. Suggestions to restructure physician workloads, develop provider satisfaction measures, and implement patient‐centered rounds provide novel additions to the PCC measurement and potential PC‐QIs. The oversight of the provider perspective is evident in a scarcity of published studies exploring both the provider and patient perspectives on health outcomes.^31^ Studies that have, however, addressed this area of research have found significant differences between provider and patient perspectives.^31^, ^32^ Physicians may feel better equipped to deliver high‐quality PCC if they are themselves satisfied and feel supported in delivering care. This finding is supported by the Institute for Healthcare Improvement's Quadruple Aim Framework, which includes improving the work life of health‐care providers as an element to optimize the health system performance.^33^ Therefore, indicators incorporating health‐care provider experience should be developed and implemented.

One participant found the PCC domains to be inadequate in addressing the social determinants of health. For example, in the domain “access to care,” there is no acknowledgment of the unique challenges to accessing care that rural or homeless populations may face. This finding is supported by past literature that has explored the understated principle of empowering patients in PCC.^34^ Pulvirenti et al^34^ describe empowerment as acknowledging and addressing the social determinants of health that affect an individual's capacity to care for themselves. Pulvirenti et al^34^ further suggest that PCC can benefit greatly from encouraging the empowerment of patients beyond the clinical relationship by addressing the social factors that may hinder an individual's ability to achieve good health. This important finding will be addressed directly, in the measurement of discriminatory care, and indirectly, through data linkage at the analysis phase prior to reporting PCC measurement.

There were some mixed views on the importance of and usefulness of PROMs and PREMs. However, it is important to highlight that participants emphasized the importance of patient feedback. Reluctance from clinicians to integrate PROMs in practice is a finding from this study as well as others.^35^ Despite this, PROMs have been found to guide treatment, shared decision making, and self‐management, and to aid clinicians in the provision of PCC.^35^

A final implication of this study is the need for future collaboration and support from authoritative entities in health‐care systems. The development of PC‐QIs serves no purpose if target audiences do not perceive the PC‐QIs as valuable for improving the quality of care. Participants' suggestions to collaborate with accreditation and professional bodies will be key in the future phases of the PC‐QI project as the goals of the project shift from developing PC‐QIs to implementation of the PC‐QIs across health‐care settings. The development of appropriate incentives for the adoption and improvement of PCC can be supported by entities that are already involved in auditing standards of practice in health care. However, rewarding physicians for meeting certain standards of care is arguably a controversial suggestion. Those who disagree with such a notion might argue that physicians should be expected to uphold a standard of care without being rewarded for doing so, or that high‐quality care should simply be an expectation of physicians. Those opposed to rewarding physicians for care have also argued that doing so may actually have the opposite effect, by diminishing intrinsic motivations.^36^ However, the use of incentives may be one way to support a workforce committed to PCC and facilitate the development of a PCC culture in health care.

### 4.1 Strengths and limitations

Although the Donabedian model provides a conceptual framework for examining health services and evaluating the quality of care, there are limitations to this framework and, thus, to the PCC domains framework that is modeled in a similar fashion. The most prominent limitation of the Donabedian framework involves the complications that arise when distinguishing between structure, process, and outcome domains. Previous studies, for example, have found that domains regarding communication may be considered either a structure domain or a process domain, depending on the context of communication.^37^ Understanding whether a given domain is considered a structure or process domain is often dependent on the target audience, as well as whether the domain is considered on a long‐term or short‐term horizon.^38^ This limitation was evident in the current study in participants' uncertainties regarding the categorization of specific domains into one of structure, process, or outcome. Consequently, the placement of each domain within the PCC framework should not be taken as absolute. Rather, it should be acknowledged that a single domain may serve a purpose in more than one area of the PCC framework. Nonetheless, this framework encouraged higher‐level discussion regarding the areas of PCC that were relevant to the experiences of clinician‐scientists, PCC researchers, and data experts alike.

While a strength of this study lies in the experience and expertise of the study participants in PCC measurement, because of the niche expertise that was required of participants within this study, selection bias is a limitation that requires consideration. Word‐of‐mouth referrals were utilized as a means of securing experts within the PCC realm, and this may have led to uniformity in perspectives. Patient voices were also not included in this study, but they are key stakeholders in the implementation of PCC. To mitigate this potential limitation for the project as a whole, a future consensus panel involving patients and clinician‐scientists will be utilized to develop and refine the PC‐QIs.

## 5 CONCLUSIONS

Patient‐centered care is known to have the potential to improve health outcomes, and adopting and improving PCC are in the interest of patients, health‐care providers, and health‐care systems.^7^, ^8^, ^9^, ^10^, ^11^, ^12^, ^13^, ^14^, ^15^, ^16^, ^17^, ^18^ Health‐care systems across Canada and in other countries have recognized PCC as a priority in the future of their health‐care systems.^1^, ^2^, ^3^, ^4^, ^5^, ^6^, ^7^, ^8^, ^9^, ^10^, ^11^, ^12^, ^13^, ^14^ Although PCC is a sought‐after model of care, metrics to measure the improvement of the quality of PCC delivered in Canada are lacking. This study outlines the perspective of ultimate end users of the metrics to monitor and improve PCC.

Clinician‐scientists and quality improvement experts suggested various future directions to improve the feasibility and impact of future PC‐QIs. The PCC domains will be useful when developing PC‐QIs, and a feasible number of PC‐QIs must be established. This study also offers suggestions to improve the PCC measurement. Along with the input of patients, families, and information from the environmental scan and scoping review already conducted, this study adds to the multiple perspectives regarding the PCC measurement upon which to begin to develop PC‐QIs. If PCC is strategically implemented using PC‐QIs, then the delivery of PCC can be optimized, and areas of improvement that are relevant to patients, family caregiver, health‐care providers, and quality improvement experts can be addressed.

## FUNDING

This study received funding from the M.S.I. Foundation and the Canadian Institutes of Health Research for the conduct of this research project (from study design to dissemination). Kimberly Manalili receives funding from the Ward of the 21^st^ Century, Cumming School of Medicine, University of Calgary, for the conduct of this research project (from study design to dissemination). Sadia Ahmed is a recipient of the 2018 Alberta SPOR Graduate Studentship in Patient‐Oriented Research, which is jointly funded by Alberta Innovates and the Canadian Institutes of Health Research. The funders played no role in study design; collection, analysis, and interpretation of data; writing of the report; or the decision to submit the report for publication

## CONFLICTS OF INTEREST

The authors report no conflict of interest.

## AUTHOR CONTRIBUTIONS

Conceptualization: Maria J Santana, Kimberly ManaliliFormal analysis: Andrea Djurkovic, Sadia Ahmed

Funding acquisition: Maria J Santana

Writing – original draft preparation: Andrea Djurkovic, Sadia Ahmed

Writing – review and editing: Sadia Ahmed, Andrea Djurkovic, Kimberly Manalili, Balreen Sahota, Maria J Santana

All authors have read and approved the final version of the manuscript.

Maria Santana had full access to all of the data in this study and takes complete responsibility for the integrity of the data and the accuracy of the data analysis.

## TRANSPARENCY STATEMENT

The corresponding author (Maria Santana) affirms that this manuscript is an honest, accurate, and transparent account of the study being reported; that no important aspects of the study have been omitted; and that any discrepancies from the study as planned (and, if relevant, registered) have been explained.

## Supporting information

Data S1

Data S2

## Data Availability

This study analyzes qualitative data, and the participants did not consent to have their full transcripts made publicly available.
