# Supplementary material for: A qualitative study on measuring patient‐centered care: Perspectives from clinician‐scientists and quality improvement experts
Source: Health Sci Rep. 2019 Nov 4;2(12):e140. doi: 10.1002/hsr2.140 (PMC6920695; doi:10.1002/hsr2.140)
Supplement: Supplementary file 1 — Data S1 [file HSR2-2-e140-s001.docx]

**Appendix 1.** Overall domains included in the Framework.^22^

| Structure | Process | Outcome |
| --- | --- | --- |
| S1. Creating a PCC culture | **P1.** Cultivating Communication | **O1.** Access to care |
| S2. Co-designing the development and implementation of educational programs | **P2.** Respectful and compassionate care | **O2.** Patient-Reported Outcomes (PROs) |
| S3. Co-designing the development and  implementation of health promotion  and prevention programs | **P3.** Engaging patients in managing their care | **O3.** Healthcare service utilization as outcomes of care |
| S4. Supporting a workforce committed to PCC | **P4.** Integration of care | **O4.** Cost of care |
| S5. Providing a supportive and accommodating PCC environment |  |  |
| S6. Developing and integrating structures to support health information technology |  |  |
| S7. Creating structures to measure and monitor PCC |  |  |
