# Supplementary material for: A qualitative study on measuring patient‐centered care: Perspectives from clinician‐scientists and quality improvement experts
Source: Health Sci Rep. 2019 Nov 4;2(12):e140. doi: 10.1002/hsr2.140 (PMC6920695; doi:10.1002/hsr2.140)
Supplement: Supplementary file 2 — Data S2 [file HSR2-2-e140-s002.docx]

**APPENDIX 2: Semi-Structured Interview Guide**

INTERVIEW GUIDE: Patient-Centered Quality Indicator Development Research

- Patient-Centered Care (PCC) is defined as “care that is respectful and responsive to individual patient preferences, needs and values, and ensuring that patient values guide all clinical decisions.”

*- Refer to PCC framework*

What is a Patient-Centered Quality Indicator (PC-QI)? “*A PC-QI* is *the unit of measurement of healthcare system performance, that quantifies what matters to patients and families, and to any individual who is in contact with healthcare services.*”

**PRE-INTERVIEW QUESTION**

1) Does this definition make sense to you? How would you define “a quality indicator for patient-centered care”?

**INTERVIEW QUESTIONS – *briefly introduce PCC domain and ask questions 2-7 domains***

- 2)  In general, do these domains make sense to you?
- 3)  Have you seen these domains in practice?
- 4)  Are these domains feasible?
- 5)  What are some of the barriers to implementing these domains? Does any of the domains present specific barriers? What are the changes required to overcome these barriers?
- 6)  In terms of reporting results in annual performance dashboards, is reporting feasible?
- 7)  Annual reports help to monitor PCC, what are some of the barriers to monitoring these domains?
- 8)  How do you see these domains changing PCC or impacting policies and protocols?

**FINAL INTERVIEW QUESTION**

9) Are there any domains of PCC that you would add to the list?
